# Protein-Bound Uremic Toxin Profiling as a Tool to Optimize Hemodialysis

**DOI:** 10.1371/journal.pone.0147159

**Published:** 2016-01-22

**Authors:** Sunny Eloot, Daniel Schneditz, Tom Cornelis, Wim Van Biesen, Griet Glorieux, Annemie Dhondt, Jeroen Kooman, Raymond Vanholder

**Affiliations:** 1 Nephrology Section, Department of Internal Medicine, Ghent University Hospital, Gent, Belgium; 2 Institute of Physiology, Medical University of Graz, Graz, Austria; 3 Division of Nephrology, Department of Internal Medicine, Maastricht University Medical Centre, Maastricht, The Netherlands; The University of Tokyo, JAPAN

## Abstract

**Aim:**

We studied various hemodialysis strategies for the removal of protein-bound solutes, which are associated with cardiovascular damage.

**Methods:**

This study included 10 patients on standard (3x4h/week) high-flux hemodialysis. Blood was collected at the dialyzer inlet and outlet at several time points during a midweek session. Total and free concentration of several protein-bound solutes was determined as well as urea concentration. Per solute, a two-compartment kinetic model was fitted to the measured concentrations, estimating plasmatic volume (V_1_), total distribution volume (V_tot_) and intercompartment clearance (K_21_). This calibrated model was then used to calculate which hemodialysis strategy offers optimal removal. Our own *in vivo* data, with the strategy variables entered into the mathematical simulations, was then validated against independent data from two other clinical studies.

**Results:**

Dialyzer clearance K, V_1_ and V_tot_ correlated inversely with percentage of protein binding. All Ks were different from each other. Of all protein-bound solutes, K_21_was 2.7–5.3 times lower than that of urea. Longer and/or more frequent dialysis that processed the same amount of blood per week as standard 3x4h dialysis at 300mL/min blood flow showed no difference in removal of strongly bound solutes. However, longer and/or more frequent dialysis strategies that processed more blood per week than standard dialysis were markedly more adequate. These conclusions were successfully validated.

**Conclusion:**

When blood and dialysate flow per unit of time and type of hemodialyzer are kept the same, increasing the amount of processed blood per week by increasing frequency and/or duration of the sessions distinctly increases removal.

## Introduction

Patients with chronic kidney disease retain a variety of solutes which have been classified as small water-soluble compounds [molecular weight (MW) below 500Da], middle molecules (MW>500Da), and protein-bound solutes [1].

In spite of their usually low MW, protein-bound solutes are difficult to remove by dialysis. Protein binding is likely an essential determinant, as ligand proteins often have a molecular weight above or at the borderline of the cut-off currently used in large-pore dialysis membranes. Convective strategies such as hemodiafiltration convey moderately better removal [2–4], most expressed with larger dialysis membranes and higher dialysate flows [5].

This lack of adequate removal may have important clinical consequences, since several protein-bound solutes have been linked to progression of renal failure, inflammation, vascular disease, and mortality [6–19].

During dialysis, solute removal is, however, not only influenced by clearance within the hemodialyzer. The role of solute transport between body compartments inside the patient is similarly important [20].So far, intradialytic kinetics have only been studied in depth for a minority of solutes of which only urea has been analyzed on a very extensive scale. In spite of the global use of urea as a marker of dialysis adequacy, its kinetics are not representative for other solutes such as phosphate [21–23],middle molecules like β_2_-microglobulin [24,25], and even other small water-soluble compounds such as the guanidines [26,27].

During extended 8hour dialysis, several solutes, such as urea, were found to be removed less during the second half of the session in spite of the clearance remaining the same throughout the session. With respect to the kinetics of protein-bound solutes, however, this phenomenon has as yet only been reported for indoxyl sulfate and p-cresylsulfate [28].

However, to the best of our knowledge, no direct kinetic data defining the distribution volume, number of compartments and intercompartmental transport of protein-bound solutes are as yet available, which is remarkable in view of their toxic potential.

Therefore, in this study we explored 1/ the kinetics of several protein-bound solutes; 2/ how these kinetics compare mutually and with those of urea; 3/ the likelihood of an association between protein binding and kinetic parameters possibly explaining a removal pattern; 4/ ways to optimize removal of protein-bound solutes by dialysis, based on a mathematical application of the data generated by this kinetic analysis; and 5/ the possibility of validating the developed model using independent data from other clinical studies [28–30] and our own *in vivo* samples [28–30], with the strategy variables entered into the mathematical simulations together with the obtained kinetic characteristics.

## Methods and Patients

### Calculations and kinetic modeling

**Fig 1:**
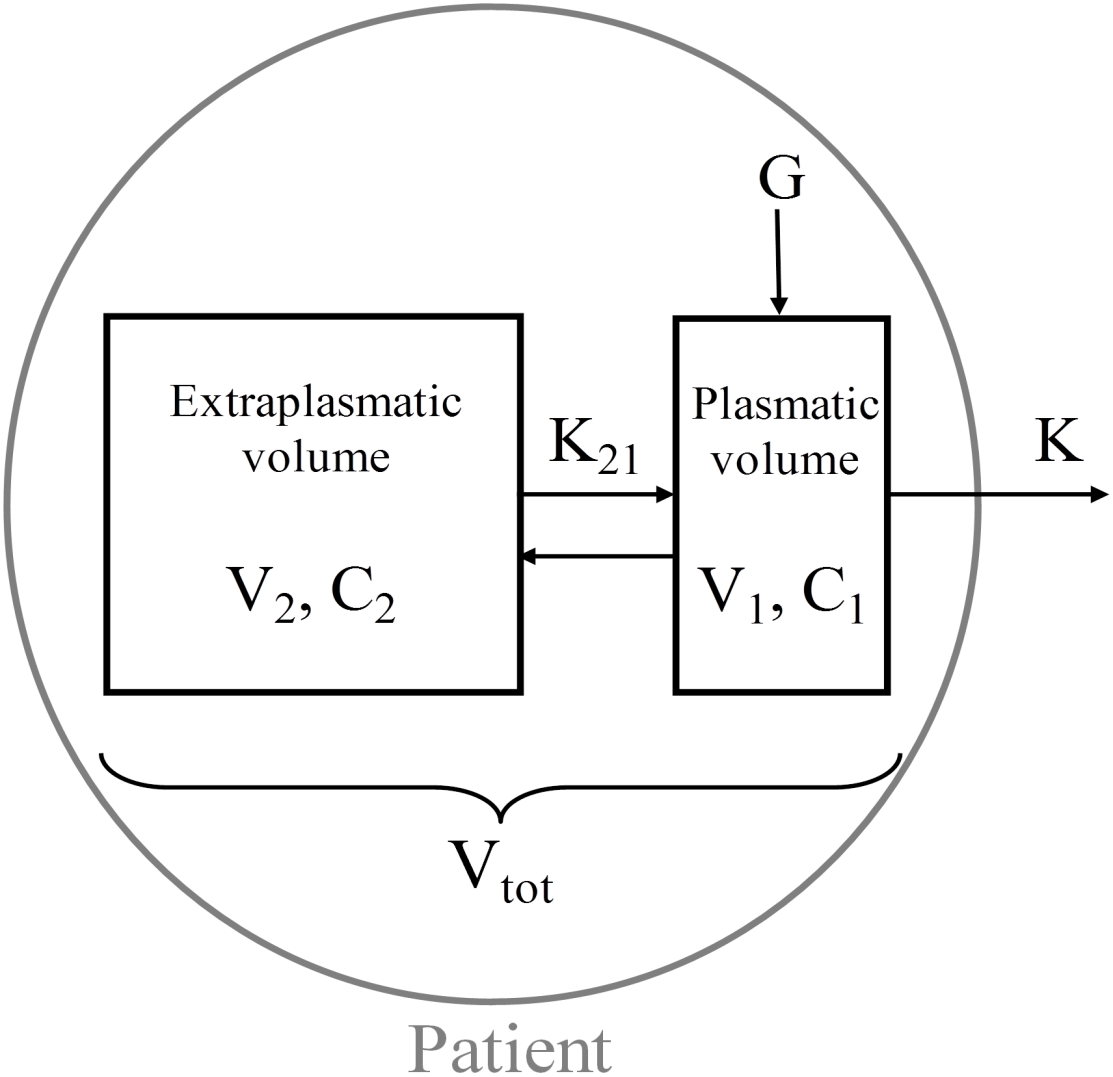
Two-compartment kinetic model. V_1_: plasmatic volume, V_2_: extraplasmatic volume, C_1_: plasmatic concentration, C_2_: extraplasmatic concentration, K: dialyzer clearance, K_21_: intercompartment clearance, G: solute generation.

The kinetic modeling approach used in this study has been previously described in depth and is based on a two-compartment model (Fig 1) [26], in which total distribution volume (V_tot_) consists of two distinct compartments: the plasmatic volume (V_1_) and the extraplasmatic volume (V_2_). The plasmatic volume is easily accessible for removal by dialysis and was assumed not to be smaller than the predialysis plasma volume, calculated as 1/13 of the total body weight (i.e. total blood volume) and accounted for predialysis hematocrit.

Urea dialyzer clearance was calculated from plasma inlet and outlet concentrations at 30 and 120 min [31], while protein-bound solute clearance was calculated from total protein-bound solute concentration in the spent dialysate and the arterial blood concentration at 30 and 120 min [32]. Since dialyzer clearances at 30 and 120 min did not differ significantly for all solutes, clearance was assumed to be constant throughout the dialysis session and was calculated as the average of both clearance values.

For each protein-bound solute, the degree of protein binding at dialysis start was derived from the relative difference between the total and free concentration of the solutes.

Presuming that removal and generation were in equilibrium, solute generation rate in the interdialytic period was assumed to be equal to the total solute removal as collected in the spent dialysate of the experimental dialysis session [33].

Ultrafiltration flow rate (Q_UF_) was taken into account to change total distribution volume over time, and was proportionally distributed over both compartments (dV_1_/dt and dV_2_/dt) based on the volume ratio of the compartments. In this way, a quick refill was assumed from the deeper tissues (extraplasmatic compartment) into the accessible plasmatic compartment. The convective solute transport is described in formula 1 and 2 in the term C.dV/dt.

The time variation of the total compartment concentration was, for a particular solute, determined by solving the mass balance equations for both compartments [26,34]:

$$\left\{ \begin{array}{l} {\text{V}}_{1}\cdot \frac{{\text{dC}}_{\text{1}}}{\text{dt}}+{\text{C}}_{1}\cdot \frac{{\text{dV}}_{\text{1}}}{\text{dt}}=\text{G}-\text{K}\cdot {\text{C}}_{1}+{\text{K}}_{\text{21}}\cdot \left({\text{C}}_{2}-{\text{C}}_{1}\right)\;(1)\\ {\text{V}}_{2}\cdot \frac{{\text{dC}}_{\text{2}}}{\text{dt}}+{\text{C}}_{2}\cdot \frac{{\text{dV}}_{\text{2}}}{\text{dt}}=-{\text{K}}_{\text{21}}\cdot \left({\text{C}}_{2}-{\text{C}}_{1}\right)\;(2) \end{array}\right.\;$$

The predialysis total concentration in both the plasmatic and extraplasmatic compartment was assumed to be equal to the measured predialysis plasma solute concentration. The kinetic model iteratively solved the mass balance equations for the complete dialysis session time, allowing the calculation of the plasmatic volume V_1_, total distribution volume V_tot_, as well as intercompartment clearance K_21_ by fitting the solution to the *in vivo* plasma concentrations measured on the samples collected during the experimental dialysis session.

### Patients

Exclusion criteria were active infection, pregnancy, unstable condition, vascular access problems, and age below 18 years. The study included ten stable chronic hemodialysis patients (two women and eight men), 69±12 years of age, 50[30;62] months on dialysis, and with a residual renal function of 2.6 [0.4;3.8] mL/min at the time of inclusion. The patients had a body weight of 75.0 [69.9;82.6]kg, hematocrit of 37.2±4.2% and a total plasma protein concentration of 61.4±6.4g/L. The study was performed during a single midweek dialysis session, during which conventional two-needle/lumen hemodialysis was performed for 240min using high-flux dialyzers: polysulfone FX800 (n = 6) (Fresenius Medical Care, Germany), heparin-grafted polyacrylonitrile Evodial (n = 1) (Gambro, Sweden), polyethersulfone Xenium 210 (n = 1) (Baxter, USA), and polyphenylene Phylter PHF 17G (n = 1) and Phylter HF 17SD (n = 1) (Bellco, Italy) in a diffusive mode. Blood and dialysate flows were prescribed at 300 and 700mL/min, respectively, while ultrafiltration rates were set according to the needs of the patients and averaged 0.41±0.29L/h. Nine patients had a well-functioning fistula and one patient a Bard Optiflo central venous catheter (Bard, USA) as vascular access. Monthly monitoring of the access flow showed no access recirculation. The mean Kt/V_urea_, as determined immediately before the study by routine monthly assessment according to the single-pool Daugirdas formula [35], was 1.7±0.3. The study was designed according to the Declaration of Helsinki, approved by the local Ethics Committee (Commissie voor Medische Ethiek—UZ Gent—Ref 2008/081—Belgian Registration Number B67020083569), and written informed consent was obtained from all participants.

### Sampling and analysis

Based on a pilot study in two patients from whom 14 samples had been collected at various time points during dialysis, it was concluded that all solutes under study (see further) were characterized by two-compartment kinetics. Hence, in the present study, blood samples were collected from the inlet blood line at the start of dialysis and after 15, 30, 60, and 120min without slowing down the blood pump, and immediately after discontinuation of the dialysis session after slowing the blood pump to 100mL/min during 15s. In addition, two blood samples were also taken from the blood outlet line at 30 and 120min. Blood samples were immediately centrifuged (3000rpm corresponding to 1250g), after which the plasma was stored at -80°C until analysis. From the outlet dialysate line, the dialysate was sampled at 30 and 120min after the start of dialysis, and was partially sampled during the entire session using a calibrated sampling system.

Urea (molecular weight MW: 60D) was measured by standard laboratory methods. Various protein-bound solutes were determined by high-performance liquid chromatography (HPLC): p-cresylglucuronide (PCG) [MW:284D, protein binding (PB)~10%], hippuric acid (HA - 179D - PB~50%), indole acetic acid (IAA - 175D - PB~65%), indoxyl sulfate (IS - 213D - PB~90%), and p-cresylsulfate (PCS - 187D - PB~95%). To determine total concentrations, serum samples were first deproteinized by heat denaturation (95°C for 30min) [36] and filtered through a molecular filter with a cut-off of 30kDa (Centrifree Micropartition Devices, Amicon Inc, Beverly, MA) prior to HPLC analysis.To assess pre-dialysis protein binding, free fractions were determined by filtering untreated serum samples through a Centrifree^®^ filter device (Millipore Billerica, MA, USA) prior to heating[37].

The instrumentation for the HPLC analyses consisted of a Waters Alliance 2695 device (Waters, Zellik, Belgium) and two detectors in series: a Waters 996 photodiode array detector (PDA) and a Waters 2475 fluorescence detector (FLD). The separation was performed at room temperature on a reversed-phase XBridge C8 column (3.5 μm, 150 mm x 4.6 mm, Waters) with an Ultrasphere ODS guard column (5 μm, 45 mm x 4.6 mm, Beckman Instruments). The mobile phase consisted of a 50 mM ammonium formate buffer (mobile phase A, pH 3.0) and methanol (mobile phase B). HA was analyzed by UV detection at 254 nm, whereas PCG and PCS (λex = 265 nm,λem = 290 nm) and IAA and IS (λex = 280 nm, λem = 340 nm) were determined by fluorescencedetection [37,38].

Hematocrit (H) was obtained by capillary centrifugation technique, and serum total protein (TP) was analyzed according to standard methods. Protein-bound solute concentrations at time point t were corrected for hemoconcentration by a factor (F) based on TP concentration at start versus time point t: F = TP_pre_/TP_t_.

### Kinetic analysis to determine optimal removal

In our search for the optimal dialysis strategy, various simulations were performed with the calibrated kinetic model, as previously described [39]. Starting from the intradialytic and interdialytic concentrations in steady state on a three times 4 h/week dialysis schedule (reference), intradialytic and interdialytic concentrations were calculated after mathematically altering several key characteristics of the dialysis regimen for the average dataset emanating out of our primary kinetic analysis. A summary of the different dialysis timeframes introduced into the calculations is given in Table 1.

**Table 1:** Terms introduced to simulate different dialysis timeframes using our calibrated kinetic models.

Strategy	TT/week(h)	BV/week(L)	Q_B_(mL/min)	Q_D_(mL/min)	Q_UF_	ER(%)
3x4h_300/w	12	216	300	700	a.m.*	a.m.*
3x8h_150/w	24	216	150	700	a.m.*/2	a.m.* x 1.33 (urea) [40]
						a.m.* x 1.00 (PBS) [41]
6x2h_300/w	12	216	300	700	a.m.*	a.m.*
6x4h_150/w	24	216	150	700	a.m.*/2	a.m.* x 1.33 (urea) [40]
						a.m.* x 1.00 (PBS) [41]
3x8h_300/w	24	432	300	700	a.m.*/2	a.m.*
6x4h_300/w	24	432	300	700	a.m.*/2	a.m.*
6x8h_150/w	48	432	150	700	a.m.*/4	a.m.* x 1.33 (urea) [40]
						a.m.* x 1.00 (PBS) [41]

3x4h_300/w: thrice weekly 4 h dialysis with Q_B_300; 3x8h_150/w: thrice weekly extended (8 h) dialysis with Q_B_150; 6x2h_300/w: frequent (6 times) short (2h) dialysis with blood flow Q_B_300; 6x4h_150/w: frequent 4 h dialysis with Q_B_150; 3x8h_300/w thrice weekly extended dialysis with Q_B_300; 6x4h_300/w: frequent 4 h dialysis with Q_B_300; 6x8h_150/w: frequent extended dialysis with Q_B_150. TT: treatment time; BV: processed blood volume; ER: extraction ratio; PBS: protein-bound solutes

a.m.*: as measured in the present kinetic study during 4 h dialysis

For the strategies with a blood flow rate of 300 mL/min, the ERs (i.e. relative change in concentration from the dialyzer inlet to outlet) and dialyzer clearances were used according to the method in the first (clinical) part of the present study where blood flows of 300 mL/min were applied. For the strategies with a blood flow rate of 150 mL/min, extraction ratios, such as those entered into the model, were adapted according to a proportion factor based on data from previous studies on urea [40] and protein-bound solutes [41] (see last column, Table 1). For all strategies, the ultrafiltration rate to be entered into the model (Q_UF_ in Table 1) was calculated such that the patients experienced the same weight loss on a weekly basis as measured during the first (clinical) part of our study. For each strategy, consecutive sessions were simulated until a new steady state of pre-dialysis solute concentration was reached, assuming that the deviation for two consecutive sessions would remain below 1%.

The various dialysis strategies were evaluated by comparing total solute removal (TSR) as calculated during the first session with the new strategy, so not yet in steady state; for frequent dialysis, the sum of the TSR from the first two sessions was used in order to reliably compare the results with the alternate day strategies [39]. Furthermore, time- averaged concentrations (TAC_1_), as calculated from the area under the concentration curve simulated for an entire week in the new steady state, and steady state predialysis concentrations (C_1_pre_) were also calculated.

### Validation of the kinetic model

Validation of the kinetic model was obtained for indoxyl sulfate and p-cresylsulfate based on the direct clinical measurements taken from the studies by Meijers et al., comparing 4*versus* 8 hour dialysis while maintaining blood and dialysate flow [28], and by Sirich et al. for higher dialysate flows Q_D_ and larger dialyzers (i.e. high versus low K_O_A-Q_D_) [29]. Validation was also obtained for all studied protein-bound solutes based on direct clinical measurements in a cross-over study from our group comparing 4 *versus* 8 hour dialysis maintaining blood and dialysate flow, and hemodialysis *versus* hemodiafiltration in 13 patients [30]. Starting from the dialysis characteristics (i.e. dialyzer clearance, ultrafiltration rate, and predialysis concentrations) as reported in those papers, our calibrated model for an average patient was used to perform the different simulations. From the obtained concentration profile, the reduction ratio was calculated and compared with the *in vivo* obtained reduction ratio.

### Statistical analysis

Normality was checked using the Shapiro-Wilk test. Data are expressed either as mean ±standard deviations in case of normal distribution or as median [25^th^ percentile; 75^th^ percentile] in case of non-normal distribution. Correlation analysis (Pearson's R) was used and additional linear regression was performed to estimate goodness of fit (R²). Statistical analyses were carried out using the Student *t* test or the non-parametric Wilcoxon signed-rank test for paired samples on normally or non-normally distributed populations, respectively. P<0.05 was taken as limit of significance.

## Results

### Calculations and kinetic model

**Fig 2:**
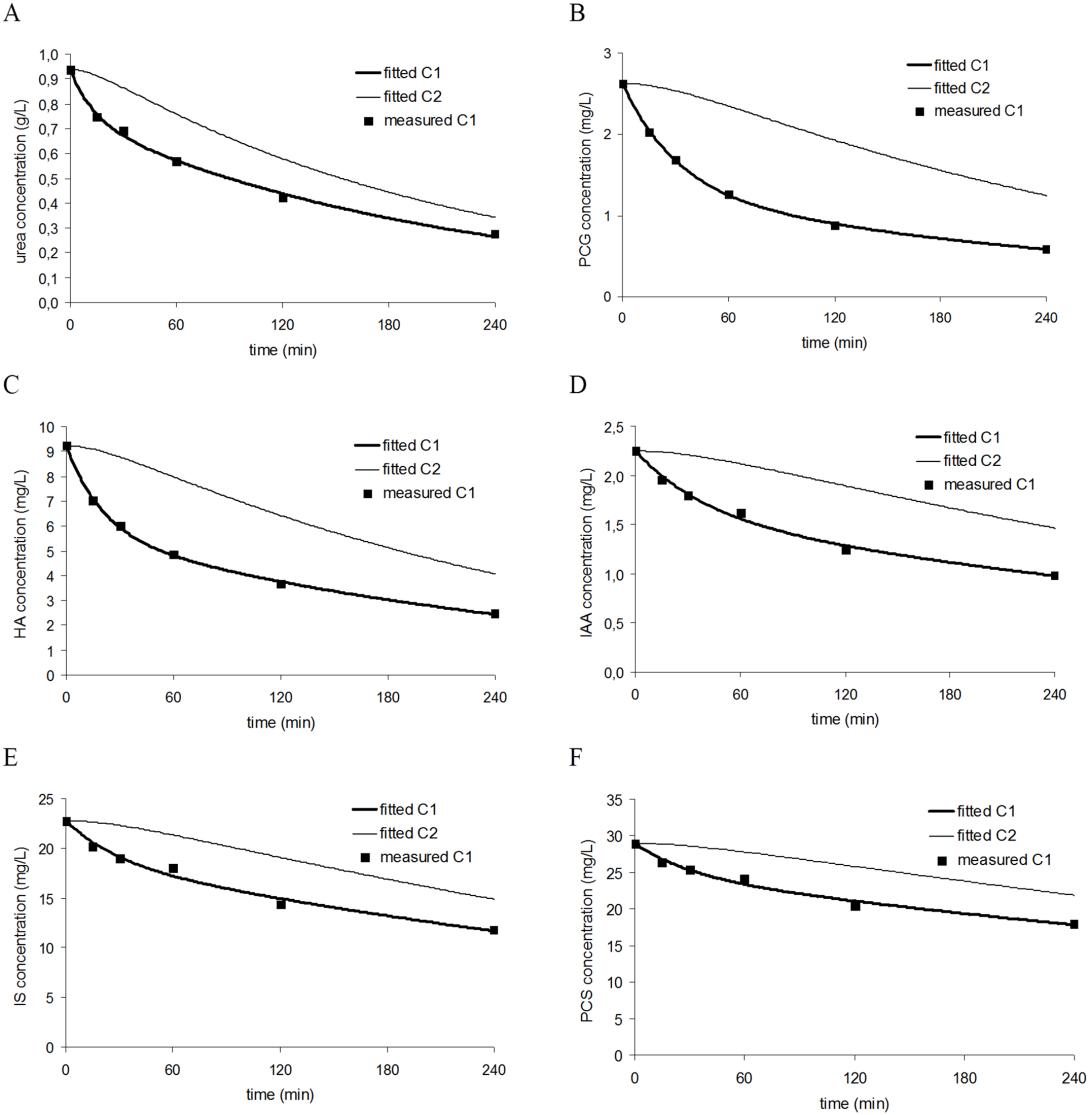
Intradialytic concentrations. Intradialytic concentrations as measured in the plasma (squares) and as simulated in the plasmatic compartment (C_1_—bold line) and extraplasmatic compartment (C_2_—thin line) of a representative patient for urea (panel A), p-cresylglucuronide (PCG) (panel B), hippuric acid (HA) (panel C), indole acetic acid (IAA) (panel D), indoxyl sulfate (IS) (panel E), and p-cresylsulfate (PCS) (panel F). All solutes show a substantial slower decline of concentration in the extraplasmatic compartment.

Starting with the assumption of two compartments (i.e. plasmatic and extraplasmatic compartment), the kinetic model was calibrated by iteratively solving the mass balance equations while fitting them to the measured concentrations during dialysis. Fig 2 illustrates, for each of the studied solutes, the measured concentrations as well as the calculated concentration profiles in the plasmatic and extraplasmatic compartment of a representative patient. All solutes show a substantially slower decline of concentration in the extraplasmatic compartment, which is the place where very likely most toxicity is exerted.

Table 2 represents the measured predialysis concentrations and the calculated parameters [protein binding (PB), solute generation rate (G), and dialyzer clearance (K)], while Table 3 shows the kinetically derived parameters [plasmatic volume (V_1_), total distribution volume (V_tot_), and intercompartment clearance (K_21_)] of the solutes under study. Solute generation was 40–1500 higher for urea as compared to protein-bound solute generation. Protein binding (PB) was different for each solute compared to the others: 13[12;15]% for p-cresylglucuronide, 47±12% for hippuric acid, 73±9% for indole acetic acid, 93[91;95]%for indoxyl sulfate, and 95[94;96]% for p-cresylsulfate. Also, all dialyzer clearances (K) were significantly different one from another (Table 2). The intercompartment clearance (K_21_) of the protein-bound solutes was 2.7 to 5.3 times lower than that of urea; there were no significant differences between protein-bound solutes.The plasmatic volume (V_1_) of the compounds with the highest protein binding, i.e. indole acetic acid, indoxyl sulfate, and p-cresylsulfate, was smaller than that of urea. Also, total distribution volume (V_tot_) of hippuric acid, indoxyl sulfate, and p-cresylsulfate was smaller than that of urea. V_tot_ of indole acetic acid was however not different from that of urea (Table 3). Altogether, the data show that a hampered removal of protein-bound solutes is the result of interactions between differences in distribution volume, intercompartment clearance and dialyzer clearance.

**Table 2:** Measured and calculated parameters for the various solutes.

Solute	PB(%)	C_pre_(mg/dL)	G(mg/min)	K(mL/min)
urea	0 ± 0	0.95 [0.90;1.28]	9.7 ± 2.6	224 ± 20
PCG	13 [12;15] ^α^	0.35 [0.26;0.79] ^α^	0.019 [0.016;0.049] ^α^	152 ± 28 ^α^
HA	47 ± 12 ^α^^β^	2.41 [1.82;4.77] ^α^^β^	0.136 [0.123;0.321]^α^^β^	132 ± 12 ^α^^β^
IAA	73 ± 9 ^α^^β^^γ^	0.21 ± 0.12 ^α^^β^^γ^	0.005 ± 0.003 ^α^^β^^γ^	52 ± 8 ^α^^β^^γ^
IS	93 [91;95] ^α^^β^^γ^^δ^	1.51 ± 0.88 ^α^^β^^γ^^δ^	0.024 ± 0.015 ^α^^γ^^δ^	27 ± 5 ^α^^β^^γ^^δ^
PCS	95 [94;96] ^α^^β^^γ^^δ^^ε^	3.06 ± 1.53 ^α^^β^^γ^^δ^^ε^	0.044 ± 0.026 ^α^^γ^^δ^	21 ± 4 ^α^^β^^γ^^δ^^ε^

PB: protein binding, C_pre_: predialysis concentration; G: solute generation, K: dialyzer clearance. PCG: p-cresylglucuronide, HA: hippuric acid, IAA: indole acetic acid, IS: indoxyl sulfate, PCS: p-cresylsulfate. P<0.05:

^α^*versus* urea;

^β^*versus* PCG;

^γ^*versus* HA;

^δ^*versus* IAA;

^ε^*versus* IS

**Table 3:** Kinetically derived parameters for the various solutes as derived from measured solute concentrations during a single midweek dialysis session.

Solute	V_1_(L)	V_tot_(L)	K_21_(mL/min)
urea	10.2 ± 5.6	32.9 ± 8.1	457 ± 175
PCG	8.2 ± 2.4	18.9 [15.7;25.2]	103 ± 47 ^α^
HA	8.4 ± 3.8	20.9 ± 4.7 ^α^	169 ± 81 ^α^
IAA	4.9 ± 1.5 ^α^^β^	24.7 ± 10.6	123 ± 46 ^α^
IS	3.7 [3.7;4.2] ^α^^β^	16.5 ± 6.5 ^α^	85 ± 72 ^α^
PCS	3.8 ± 0.9^β^^γ^	10.4 [9.8;15.1] ^α^^δ^	87 ± 44 ^α^

V_1_: plasmatic volume, V_tot_: total distribution volume, K_21_: intercompartment clearance. PCG: p-cresylglucuronide, HA: hippuric acid, IAA: indole acetic acid, IS: indoxyl sulfate, PCS: p-cresylsulfate. P<0.05:

^α^*versus* urea;

^β^*versus* PCG;

^γ^*versus* HA;

^δ^*versus* IAA.

### Correlations with protein binding and body weight

**Fig 3:**
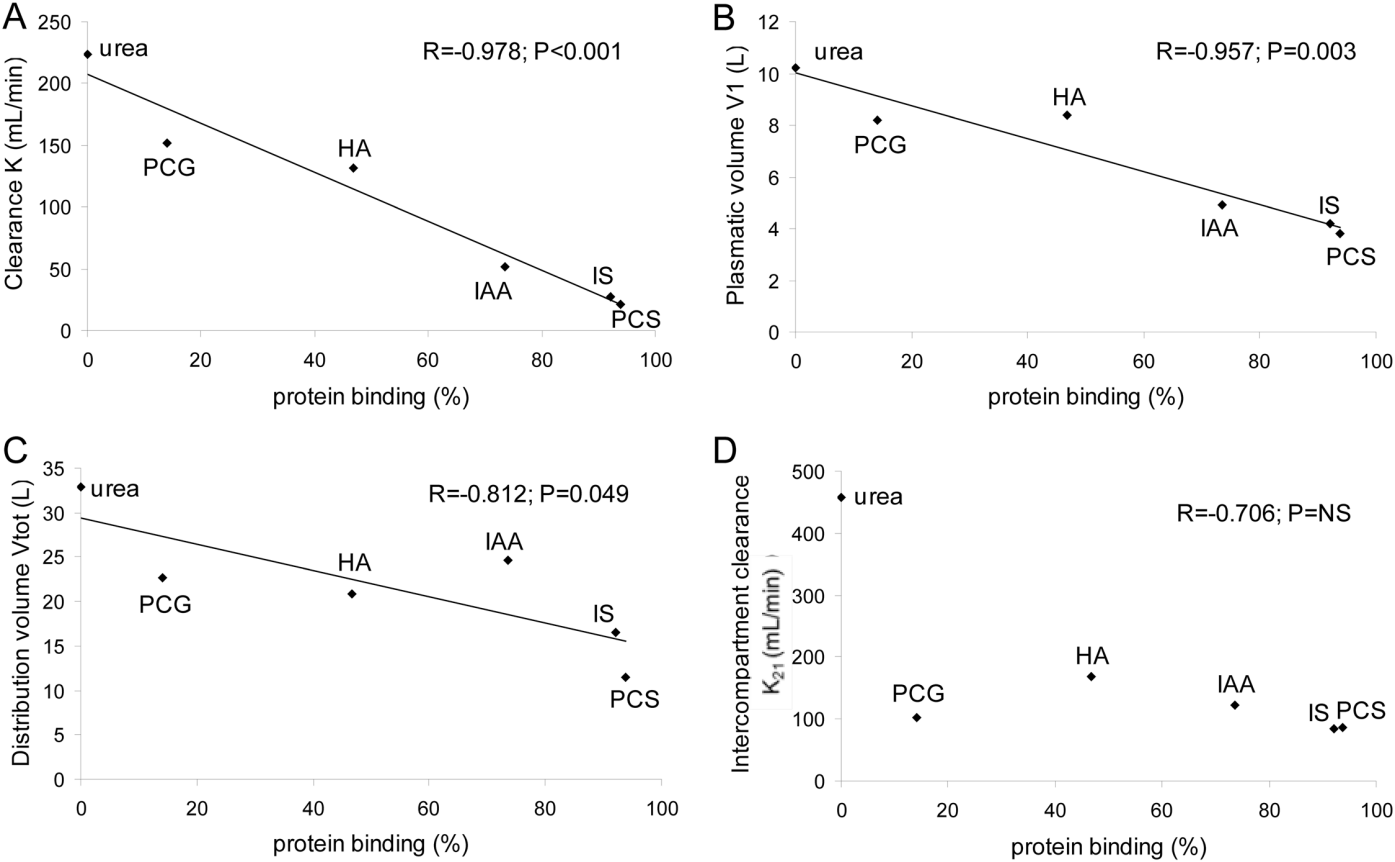
**Correlation of the percentage of protein binding:**with the dialyzer clearance K (panel A), plasmatic volume V_1_which is easily accessible for removal by dialysis (panel B), total distribution volume V_tot_ (panel C), and the intercompartment clearance K_21_ (panel D) of PCG: p-cresylglucuronide, HA: hippuric acid, IAA: indole acetic acid, IS: indoxyl sulfate, and PCS: p-cresylsulfate.

Protein binding correlated inversely with K (R = - 0.978; P<0.001), V_1_ (R = - 0.957; P = 0.003) and V_tot_ (R = - 0.812; P = 0.049) but not with K_21_ (R = - 0.706; P = NS) (Fig 3). The non significant correlation with K_21_ is to a large extent due to the low value of K_21_ for p-cresylglucuronide, in spite of its virtual zero protein binding. Without p-cresylglucuronide, the correlation coefficient was R = -0.959 with P = 0.037. No correlations were found between the kinetic parameters and the patient's body weight.

### Definition of optimal removal by kinetic analysis

Besides standard dialysis (i.e. three times 4h/week with 300mL/min blood flow), six other strategies (more frequent and/or longer and/or lower blood flow) were simulated. Table 4 reports the calculated TSR for the first dialysis with a given strategy for the solutes studied, as well as steady state TAC_1_ and predialysis concentration (C_1_pre_).

**Table 4:** Dialysis adequacy in different strategies.

	urea	PCG	HA	IAA	IS	PCS
	Total Solute Removal (TSR)					
	g	mg	mg	mg	mg	mg
3x4h_300/w	28.3	111	679	17.1	83.5	153
3x8h_150/w	33.8	126	743	18.7	88.5	163
6x4h_150/w	41.3	151	891	20.4	95.3	177
6x2h_300/w	30.9	139	843	19.1	92.2	170
3x8h_300/w	37.4	152	907	27.4	131	235
6x4h_300/w	46.4	185	1114	30.1	143	258
6x8h_150/w	51.0	201	1178	32.4	149	273
	Time Averaged Concentration (TAC_1_)					
	g/L	mg/L	mg/L	mg/L	mg/L	mg/L
3x4h_300/w	0.80	5.37	32.2	1.62	12.8	28.8
3x8h_150/w	0.62	4.54	29.0	1.47	11.9	29.1
6x4h_150/w	0.52	4.08	26.2	1.52	12.2	29.9
6x2h_300/w	0.71	4.65	28.6	1.59	12.9	30.0
3x8h_300/w	0.52	3.31	20.5	0.93	7.3	16.3
6x4h_300/w	0.40	2.63	16.1	0.84	7.0	15.6
6x8h_150/w	0.30	2.20	14.2	0.87	7.4	17.6
	Predialysis concentration (C_1_pre_)					
	g/L	mg/L	mg/L	mg/L	mg/L	mg/L
3x4h_300/w	1.13	7.26	44.3	1.89	14.5	33.0
3x8h_150/w	0.93	6.31	40.3	1.72	13.6	33.0
6x4h_150/w	0.66	4.92	31.8	1.66	13.2	32.0
6x2h_300/w	0.86	5.56	34.3	1.72	13.8	32.0
3x8h_300/w	0.83	5.11	32.1	1.14	9.0	20.6
6x4h_300/w	0.54	3.49	21.4	0.99	8.1	17.9
6x8h_150/w	0.41	2.90	18.4	0.98	8.1	19.3

Total solute removal (TSR), time-averaged concentrations (TAC_1_) and predialysis concentrations (C_1_pre_) for the various solutes studied as calculated from simulations of various dialysis strategies; TSR as calculated from the first session with the new strategy and TAC_1_ and C_1_pre_ determined once a new steady state is reached. PCG: p-cresylglucuronide, HA: hippuric acid, IAA: indole acetic acid, IS: indoxyl sulfate, PCS: p-cresylsulfate. 3x4h_300/w: thrice weekly 4h dialysis with Q_B_300; 3x8h_150/w: thrice weekly extended (8h) dialysis with Q_B_150; 6x2h_300/w: frequent (6 times) short (2h) dialysis with blood flow Q_B_300; 6x4h_150/w: frequent 4h dialysis with Q_B_150; 3x8h_300/w thrice weekly extended dialysis with Q_B_300; 6x4h_300/w: frequent 4h dialysis with Q_B_300; 6x8h_150/w: frequent extended dialysis with Q_B_150.

**Fig 4:**
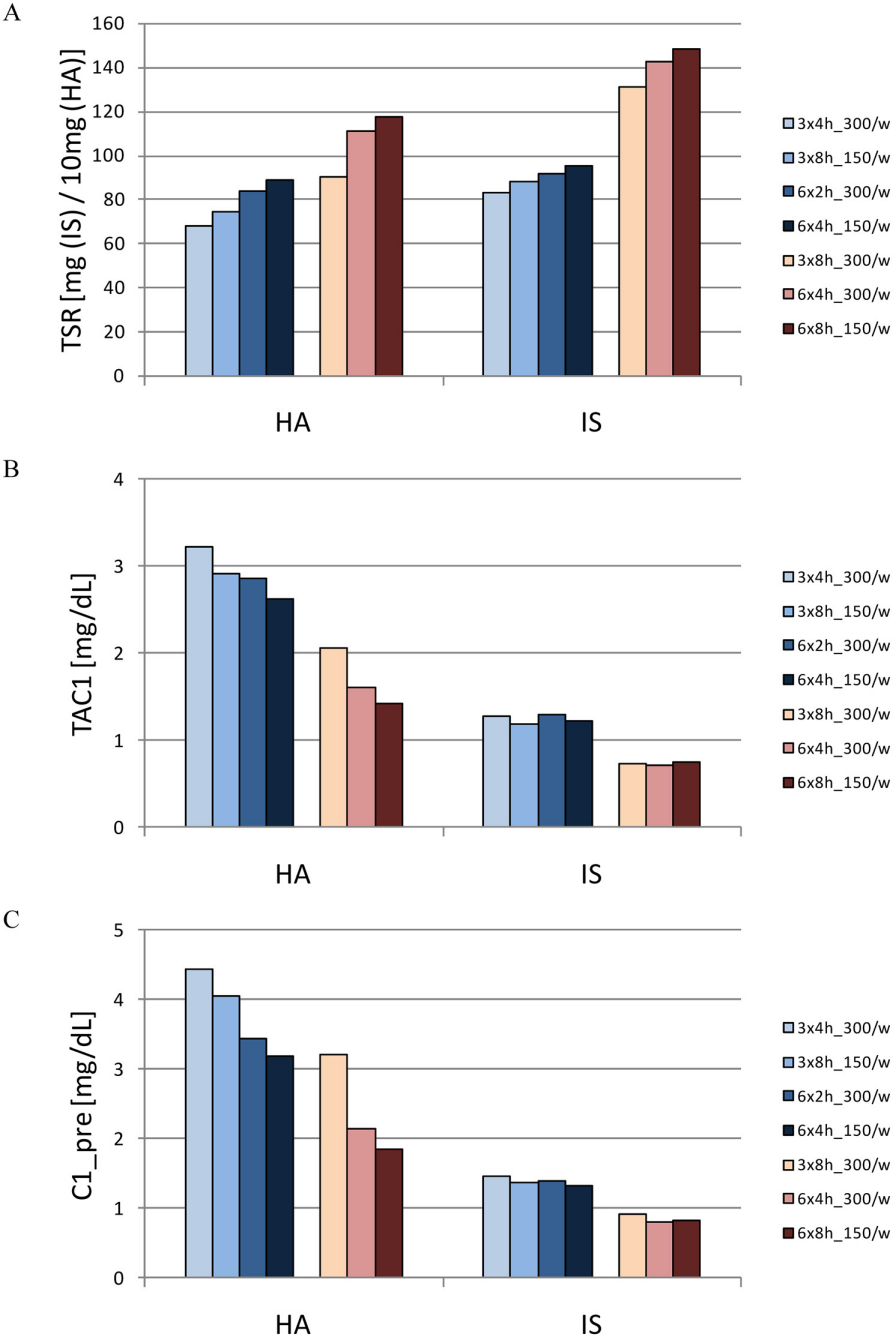
Adequacy in different dialysis strategies. Total solute removal (TSR—panel A), time-averaged concentration (TAC_1_—panel B) and predialysis concentration (C_1_pre_—panel C) of hippuric acid and indoxyl sulfate for seven different dialysis strategies. The different shades of blue and red, respectively, refer to the regimes with less and more processed blood as compared to 3x4h_300/w. The two darkest bars on the right of each series refer to the more frequent dialyses. [TSR in 10^1^mg (HA) and mg (IS); TAC_1_ and C_1_pre_ in mg/dL]. [3x4h_300/w: thrice weekly 4h dialysis with Q_B_300; 3x8h_150/w: thrice weekly extended (8h) dialysis with Q_B_150; 6x2h_300/w: frequent (6 times) short (2h) dialysis with blood flow Q_B_300; 6x4h_150/w: frequent 4h dialysis with Q_B_150; 3x8h_300/w thrice weekly extended dialysis with Q_B_300; 6x4h_300/w: frequent 4h dialysis with Q_B_300; 6x8h_150/w: frequent extended dialysis with Q_B_150].

Fig 4 illustrates the effects of different time frames on two representative solutes: hippuric acid (also representing urea and p-cresylglucuronide as solutes with no or low protein binding) and indoxyl sulfate (representing solutes with high protein binding, such as p-cresylsulfate and indole acetic acid). For the strongly bound solutes, the conclusion is straightforward: with an increase in the weekly processed blood volume from 216 to 432L, a marked increase in removal occurs whatever the timeframe imposed to reach these volumes. Between different timeframes, with either one of the two studied quantities of processed blood, the differences are not remarkable. For the solutes with lower protein binding, the difference between the most extreme regimens with regard to efficiency is of a similar order, but the effect of total processed blood is less preponderant while time and frequency seem to play a more prominent role. Among strategies with which the amount of processed blood is the same, increasing frequency seems more beneficial than increasing dialysis duration in removing solutes with low protein binding (Fig 4).

### Validation of the kinetic model

**Fig 5:**
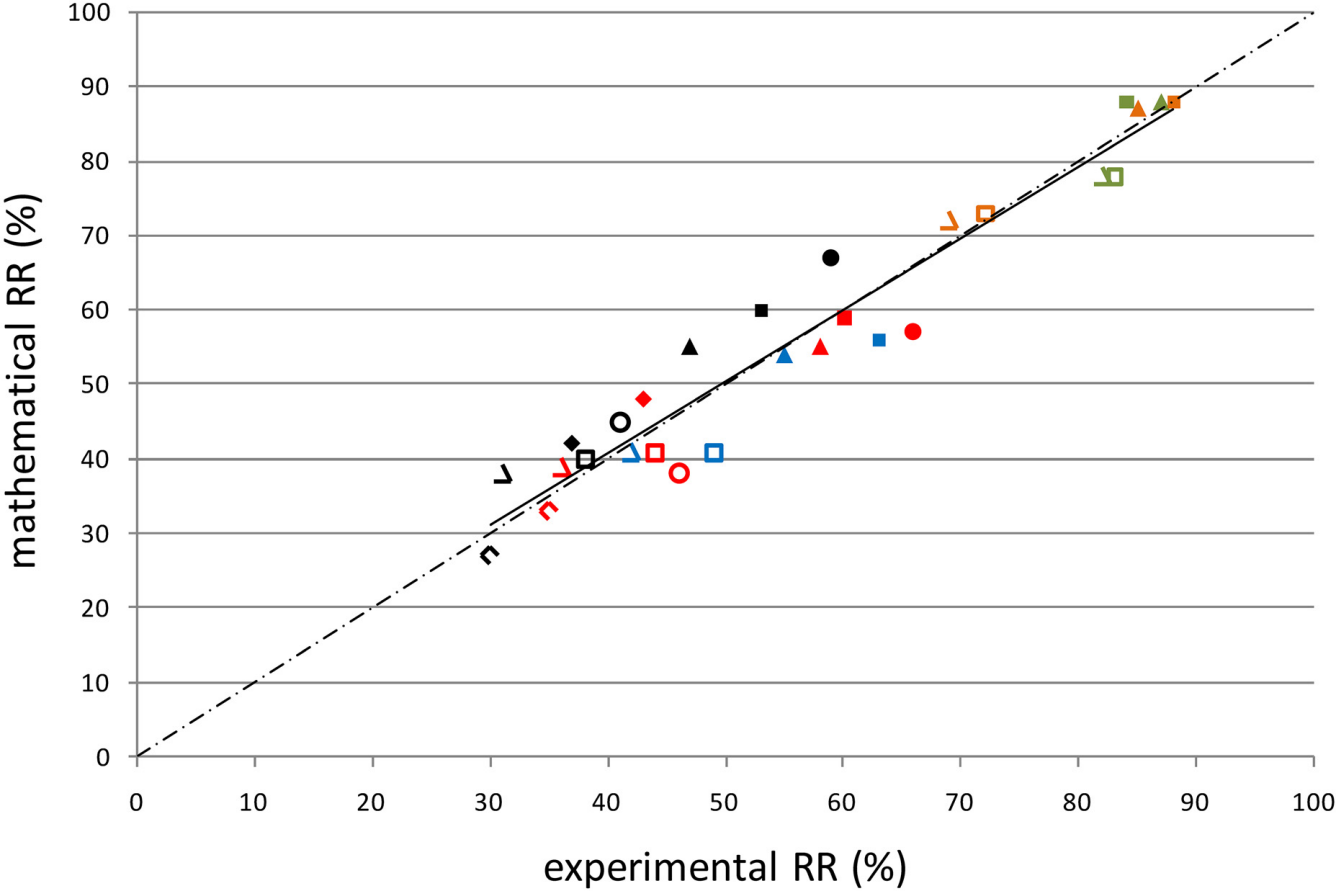
Model validation. Correlation (R = 0.942; P<0.001) between the reduction ratios as measured *in vivo* and those as derived from mathematical analyses for the different protein-bound solutes [PCG (green), HA (orange), IAA (blue), IS (red), and PCS (black)] and the different dialysis strategies [4h HD (open diamonds) and 8h HD (filled diamonds) for correlation with the study of Meijers et al.; low K_O_A (open circles) and high K_O_A (filled circles) for correlation with the study by Sirich et al.; 4h hemodialysis HD (open triangles), 8h HD (closed triangles), 4h hemodiafiltration HDF (open squares), and 8h HDF (filled squares) for correlation with our own *in vivo* data].

The calibrated model was validated by mathematical simulation of various dialysis strategies as applied in two other clinical studies [28];[29]as well as in a study of our group (cf. study [29]). The reduction ratios for indoxyl sulfate and p-cresylsulfate with standard 4h and prolonged 8h dialysis as calculated with our calibrated kinetic model differed by only 2–5% from those directly measured by Meijers et al.[28]. For the same compounds, the reduction ratios with low and high K_O_A-Q_D_ (dialyzer permeability-area coefficient—dialysate flow) dialysis as calculated with our kinetic model differed by 4–9% from those measured clinically by Sirich et al.[29].More importantly, even with a direct comparison of the results of our mathematical findings and data obtained *in vivo* with five uremic toxins (p-cresylglucuronide, hippuric acid, indole acetic acid, indoxyl sulfate, and p-cresylsulfate) and four different strategies [4h hemodialysis (HD), 8h HD, 4h hemodiafiltration (HDF), and 8h HDF while maintaining blood and dialysate flow], differences again were between 1–5% (p-cresylglucuronide), 0–3% (hippuric acid), 1–8% (indole acetic acid), 1–3% (indoxyl sulfate), and 2–8% (p-cresylsulfate)[30]. These minor deviations between measured and calculated reduction ratios result in a strong mutual correlation (R = 0.942; P<0.001) (Fig 5). Taken together, the direct *in vivo* data thus strongly corroborate the findings obtained by kinetic modeling.

## Discussion

The present study was undertaken to 1/ describe the kinetics of protein-bound solutes during dialysis, 2/ compare these kinetics among protein-bound solutes and with those of the standard marker urea, 3/ unravel the mechanisms how protein binding is related to dialytic kinetics and removal patterns of protein-bound solutes, 4/ find the optimal dialysis strategy for removal of these solutes, and 5/ validate our kinetic model using independent clinical data as well as our own *in vivo* data.

The main findings of this study are that 1/ protein-bound solutes have kinetic characteristics that are substantially different from those of urea, but there are also marked differences among protein-bound solutes *per se*, 2/ dialyzer clearance and distribution volume show an inverse relation with the percentage of protein binding, 3/ for the strongly bound solutes, adequacy is, for each specific type of dialyzer, mainly determined by the weekly amount of processed blood, 4/ for the solutes with lower protein binding, adequacy is, next to the amount of processed blood, also determined by dialysis frequency and to a lesser extent by dialysis length, and 5/ the validation studies show that our kinetic model offers a representative reproduction of real-life clinical dialysis for all protein-bound solutes that we assessed.

Several studies already stressed that urea kinetics are not congruent with those of other solutes like other small water soluble compounds [21–23,26,27]as well as middle molecules [24,25,42].We now add to these findings by determining that there is also a discrepancy between urea kinetics and those of protein-bound solutes, mathematical proof of what was previously suggested by a correlation study showing a dissociation between removal of urea and indoxyl sulfate [43]. In addition, kinetics are also substantially divergent among individual protein-bound solutes, a trend that was also observed for small water soluble compounds [21–23,26,27]and middle molecules [44]. Even among derivatives of the same mother compound (p-cresylsulfate and p-cresylglucuronide) marked differences are seen. Previously, we also found differences in toxicity pattern [45] stressing that one cannot automatically extrapolate conclusions about one solute to another, even if they have a closely related biochemical structure and origin. However, when taking into account the strongly bound proteins, only (PB > 90%), the kinetics are fairly similar, and considering one single molecule seems fairly representative of the whole group, independent of the origin. Of note, for these solutes, ample proof is available that they exert strong biological activity [46], and therefore, knowledge of their respective kinetics is of utmost importance when trying to increase their dialytic removal. First, dialyzer clearance is markedly low for the strongly bound solutes since only the free fraction can pass the dialyzer membrane by diffusion. Second, the intercompartment clearance (K_21_), which is representative of the shift from outside the plasma (e.g. the cell and tissues) into the plasma, is markedly low for these strongly bound solutes (Table 3). These findings point to a hampered removal from the intracellular or intramuscular level, whereas it is likely that most if not all the biochemical (toxic) effects of these compounds are exerted in that compartment. Our two-compartmental models with low intercompartment clearances for the strongly bound solutes also corroborate the previous findings by Meijers et al.[28]. The latter, applying a one compartment model, described retardation of indoxyl sulfate and p-cresylsulfate transport inside the patient, and with it less pronounced concentration decreases during the second half of an extended dialysis session, as if the volume of this single compartmental model (i.e. the apparent volume in Meijers et al.) increased during the second half of the session. An alternative explanation is, however, that for protein-bound solutes, the two-compartmental model better reflects the evolution within the patient. When using a single compartmental model on our present 4 hour clinical data, a significant overestimation of fitted intradialytic concentrations is found during the first half, followed by an underestimation during the second half of the session. Furthermore, simulating the various dialysis strategies as reported by Meijers et al.[28] and Sirich et al.[29] using the for IScalibrated one-compartment model, resulted in reduction ratios deviating up to 26–58% and 24–33% as compared to those measured by Meijers et al. and Sirich et al., respectively, in a clinical setting.

Our data also stress that the removal pattern of protein-bound uremic solutes is markedly different from that of other uremic toxins, even if they have a similar molecular weight as the protein-bound solutes themselves (small water soluble compounds) or as the carrier proteins to which those solutes are bound (middle molecules). Thus, with regard to dialysis, as much as to toxicity, protein-bound uremic toxins should be considered as separate entity. Furthermore, this is also the case considering residual renal function (RRF) since protein-bound solutes are mostly excreted in the tubules, such that RRF, a surrogate for glomerular filtration rate, may not equal the tubular excretion. In the present study, patients were nearly anuric and RRF was not incorporated as a separate parameter in the kinetic model. However, for patients with substantial RRF, an extra clearance parameter with impact on the plasmatic volume should be included.

There is less removal of protein-bound solutesthan non-bound solutes of similar molecular weight [38,47,48]. Meyer et al. previously found that using dialyzers with higher mass transfer area coefficient in combination with higher dialysate flow rates is a successful strategy to increase protein-bound solute dialyzer clearance without the need to change the dialysis time schedule [48]. Our data now indicate that, for a fixed type of dialyzer, both extended and frequent dialysis with maintenance of the same amount of processed blood per session as in standard dialysis only results in limited decreases in solute concentration for the strongly protein-bound compounds; time-averaged indoxyl sulfate concentrations were reduced by 0.1mg/dL while this is 0.33mg/dL for the highest dose of AST-120, a peroral sorbent [49].However, with frequent extended or frequent high-flow dialysis resulting in large amounts of processed blood, time-averaged concentrations could, according to our calculations, be reduced to a higher degree (i.e. 0.51mg/dL for TAC of indoxyl sulfate).

For the solutes with lower protein binding, adequacy is, next to the amount of processed blood, also determined by dialysis frequency and to a lesser extent by dialysis length. This finding corroborates with the formula of the Hemodialysis Product (HDP) as described by Scribner et al as a better index of dialysis adequacy than Kt/V_urea_ (i.e. HDP is much higher for frequent than for prolonged dialysis for the same number of dialysis hours per week)[50].

The findings describing an impact of more frequent and extended dialysis on these protein-bound solutes, should be considered with the results of clinical studies on the impact of changes in dialysis timeframe on dialysis. Whereas a controlled study by Chertow et al. showed a survival advantage of frequent dialysis [51], the one by Rocco et al found no advantage for frequent extended dialysis [52]. The latter study was however skewed by the need to bring down the number to treat twice, probably leading to decreased statistical power.

Independent validation of research results has become one of the main tools of modern research. This has especially been emphasized in the context of 'omics' research [53], but is valid for many other areas in clinical science. We therefore thought it important to test the kinetic model as developed in the present study against results of clinical studies performed by independent researchers [28,29] besides our own *in vivo* data [30]. The fact that the results of two other studies [28,29] could be predicted on the basis of other analytical techniques only underscores that our kinetic model is robust and can be applied in many conditions. Even more importantly, in a study in our own hands comparing the removal pattern of protein-bound solutes with four different timeframes in an independent population, again the mathematical data were corroborated. The strong correlation between measured and mathematically derived reduction ratios, as well as the limited deviations among them, are proof of the validity of our calibrated kinetic model.

Therefore, this validation also supports that the findings of the calculations for different strategic frames can be extrapolated to clinical reality. Hence, our kinetic model could be helpful in finding novel strategies without being forced to submit patients to multiple blood samplings and multiple different dialysis strategies, and without undertaking numerous laborious analytical determinations.

In conclusion, protein-bound solute kinetics is complex and specific for each compound. While dialyzer clearance differs depending on the percentage of protein binding, differences in intercompartment shifts further decrease removal possibilities. For a fixed type of hemodialyzer, while maintaining blood and dialysate flows unmodified, protein-bound solute removal is enhanced by increasing the amount of processed blood per week by increasing frequency and/or duration of the dialysis session, especially for the removal of indoxyl sulfate and p-cresylsulfate, both known to exert toxicity. Our kinetic model can be further used to predict removal and concentrations of these solutes with other strategies.
